# Treatment Outcomes of Infectious and Non-infectious Acute Exacerbation of Myositis-Related Interstitial Lung Disease

**DOI:** 10.3389/fmed.2021.801206

**Published:** 2022-03-07

**Authors:** Hyun Lee, Sung Jun Chung, Sang Hyuk Kim, Hayoung Choi, Youlim Kim, Tai Sun Park, Dong Won Park, Ji-Yong Moon, Sang-Heon Kim, Tae Hyung Kim, Ho Joo Yoon, Jang Won Sohn

**Affiliations:** ^1^Division of Pulmonary Medicine and Allergy, Department of Internal Medicine, Hanyang University College of Medicine, Seoul, South Korea; ^2^Division of Pulmonology and Critical Care Medicine, Department of Medicine, Samsung Medical Center, Sungkyunkwan University School of Medicine, Seoul, South Korea; ^3^Division of Pulmonary, Allergy, and Critical Care Medicine, Department of Internal Medicine, Hallym University Kangnam Sacred Heart Hospital, Hallym University College of Medicine, Seoul, South Korea; ^4^Division of Pulmonary, Allergy and Critical Care Medicine, Department of Internal Medicine, Konkuk University Hospital, School of Medicine, Konkuk University, Seoul, South Korea

**Keywords:** myositis, inflammatory myopathy, polymyositis, dermatomyositis, interstitial lung disease, acute exacerbation, respiratory failure

## Abstract

**Introduction:**

Although respiratory infections are common causes of acute respiratory failure (ARF) in patients with myositis-interstitial lung disease (ILD), limited data are available regarding the treatment outcomes by the etiologies of acute exacerbation (AE) of myositis-related ILD (infectious vs. non-infectious). Our study aimed to investigate the treatment outcomes of AE in patients with myositis-related ILD focused on the infectious etiology.

**Methods:**

A single center-based retrospective cohort was performed at Hanyang University Hospital between January 2000 and December 2018. A total of 36 patients with AE of myositis-related ILD were consecutively included. The exposure was the etiologies of AE in myositis-related ILD, and the outcome was in-hospital mortality. The infectious etiology was defined as confirmation of bacteria, virus, or fungus in samples obtained from the respiratory tract.

**Results:**

Among the 36 patients, 17 were diagnosed with infectious AE. The overall in-hospital mortality rate of AE was 47.2%. Although the mortality rate in patients with infectious AE was lower (41.2%) than in those with non-infectious AE (52.6%), this difference was not statistically significant (*p* = 0.724). A survival analysis showed no significant difference in mortality between patients with infectious AE versus those with non-infectious AE [risk ratio = 0.78, 95% CI = 0.38–1.59].

**Conclusion:**

Our study showed that infectious AE is an important cause of mortality in patients with myositis-related ILD, showing a similar risk of mortality as non-infectious AE.

## Introduction

Idiopathic inflammatory myopathies (IIMs), mainly dermatomyositis and polymyositis, are autoimmune diseases involving skeletal muscle inflammation of unknown cause (1). Extra-skeletal muscle involvements commonly occur in IIM, and lung involvement is known as myositis-related interstitial lung disease (ILD) (2). With advances in the treatment of myositis, the prognosis of patients has been improved (3). However, the mortality rate of patients with myositis-related ILD who develop acute respiratory failure (ARF) is still very high (4). Unfortunately, the prognosis of acute exacerbation (AE) of myositis-related ILD has been reported inconsistently, and there is no definite consensus on diagnosis and treatment (5, 6).

Infectious conditions, as well as non-infectious conditions, such as idiopathic AE of ILD and drugs, can cause ARF in patients with IIM (7). The definition of AE of ILD has been the most well-studied in idiopathic pulmonary fibrosis (IPF) (8). The conventional definition of AE of IPF considered infection etiologies as a reversible condition and excluded it (9). However, recent studies suggest that infection may play a role in the AE of IPF (10). Consequently, the new definition of AE of IPF was divided into AE with or without a trigger (8).

Since patients with myositis commonly use immunosuppressive drugs, respiratory infections can cause ARF in patients with myositis-related ILD (11). However, due to the rarity of this disease, it is unknown whether infectious ARF can be applied to patients with myositis-related ILD, as in IPF. Accordingly, our study aimed to investigate the treatment outcomes of ARF in patients with myositis-related ILD focused on the etiologies, i.e., infectious and non-infectious causes.

## Methods

### Study Design and Population

**Figure 1:**
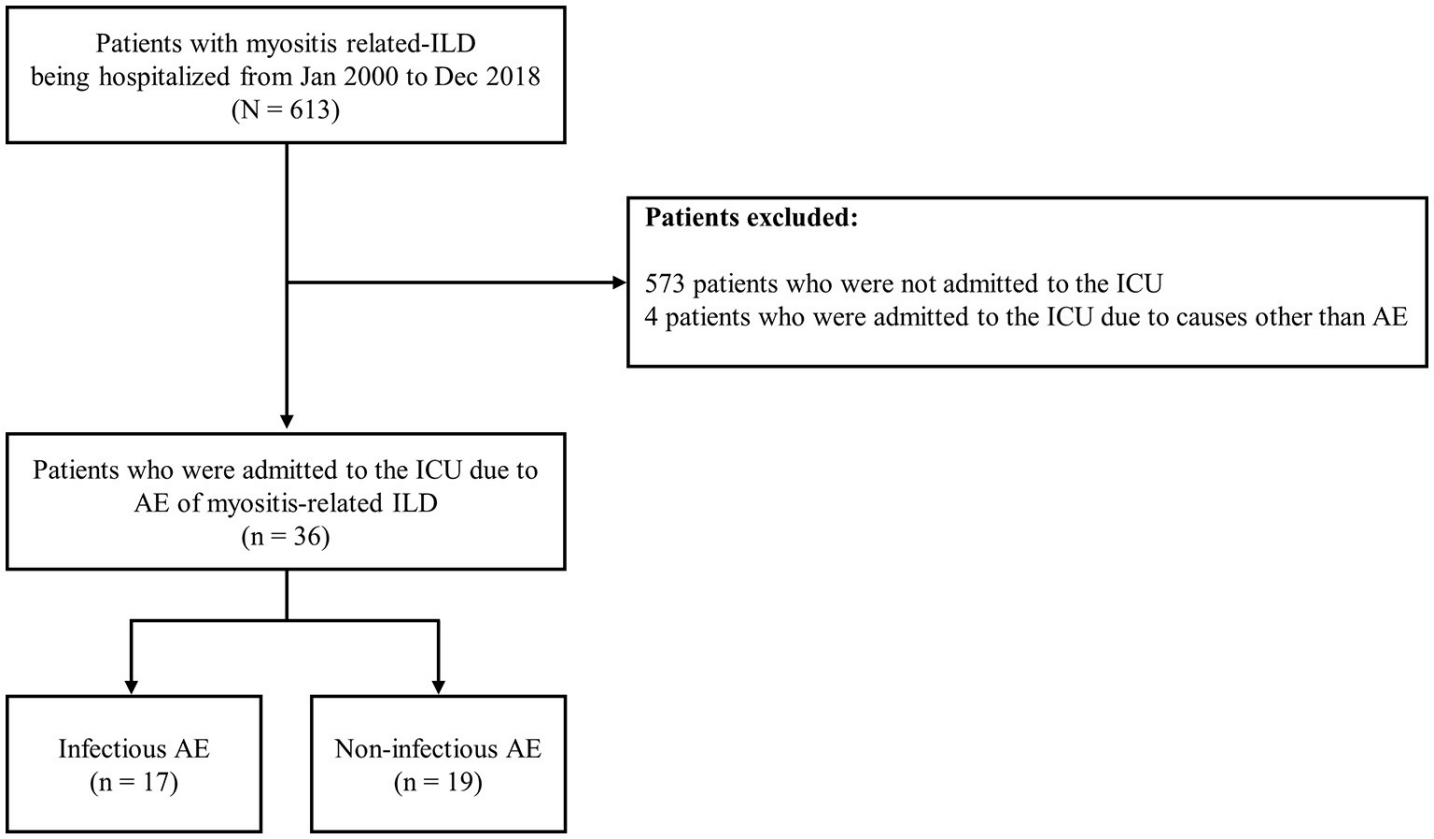
Flowchart of patient inclusion. ILD, interstitial lung disease; ICU, intensive care unit; AE, acute exacerbation.

A single center-based retrospective cohort study was performed at Hanyang University Hospital between January 2000 and December 2018. Of 613 patients with myositis-related ILD being hospitalized during the study period, we excluded 573 patients who were not admitted to the intensive care unit (ICU) and 4 patients who were admitted to the ICU due to causes other than AE. As a result, 36 patients with AE of myositis-related ILD were consecutively included (Figure 1). IIM was defined using previous criteria (12). The presence of ILD was determined using the high-resolution computed tomography (HRCT) findings.

This study was conducted following the Helsinki declaration, and ethics approval was obtained from the Institutional Review Board (IRB) of Hanyang University Hospital (IRB number: HYUH 2020-04-056-002). The need for written informed consent was waived because this was a retrospective cohort study.

### Exposure and Outcome

The exposures were etiologies of AE in patients with myositis-related ILD. We defined AE of myositis-related ILD as (1) acute worsening <1 month; (2) new ground-glass or consolidation on HRCT; and (3) no evidence of left arterial hypertension on echocardiography (8). The etiology was classified into infectious and non-infectious. To assess the etiology of AE, all patients underwent bronchoalveolar lavage (BAL) within the first 24 h of admission. Infectious etiology was defined as confirmation of bacteria, virus, or fungus in samples, such as BAL, obtained from the respiratory tract. Regardless of the etiologies, all patients received systemic corticosteroids. All patients with infectious AE received appropriate antibiotics that covered the microorganism identified. The main outcome was in-hospital mortality.

### Data Collection

We reviewed patients' electronic medical records to collect the following data: baseline demographics [age, sex, body mass index, and smoking status], inflammatory myopathy subtype, autoantibodies, maintenance immunosuppressive medications, lung function, comorbidities, and the clinical manifestation of AE (age at AE, disease severity, radiologic features, time from ICU admission to the BAL, laboratory findings, medications for treating AE, CU length of stay, and in-hospital mortality). Disease severity was assessed using the ratio of arterial oxygen partial pressure to fractional inspired oxygen (PF ratio), acute physiology and chronic health evaluation (APACHE) score, and the need for mechanical ventilation. According to the American Thoracic Society (ATS)/European Respiratory Society (ERS), pulmonary function was measured using a spirometer (13). The percentage-predicted values of forced expiratory volume in 1 s (FEV_1_) and forced vital capacity (FVC) were measured using the Korean formula (14). Regarding comorbidities, hypertension, diabetes mellitus, and pulmonary hypertension were evaluated by a chart review.

### Statistical Analyses

Continuous variables were presented as median with interquartile range (IQR), and categorical variables were presented as percentages. The value of *p* was analyzed for continuous variables by the Wilcoxon Rank-Sum test and for categorical variables by the chi-squared test and Fisher's exact test depending on the event numbers. The Kaplan–Meier curve was drawn using the “survival” and “survminer” package of R. The risk ratio (*RR*), 95% confidence interval *(CI)*, and *p* were calculated using the “fmsb” package of R. All statistical analyses were performed using R 4.0.3 (R Foundation for Statistical Computing, Vienna, Austria).

## Results

### Baseline Characteristics

The baseline characteristics of the patients are described in Table 1. At the diagnosis of myositis-related ILD, the median (IQR) of age was 50 (39–59) years, and 69.4% were women. Most of the patients had dermatomyositis (86.1%). The proportion of those with dermatomyositis was lower in patients who developed infectious AE than in those who developed non-infectious AE (70.6 vs. 100%, *p* = 0.039).

**Table 1:** Baseline characteristics of patients with myositis-related interstitial lung disease (ILD).

	**Total (*n* = 36)**	**Non-infectious AE (*n* = 19)**	**Infectious AE (*n* = 17)**	***p*-value**
**Socio-demographic profile**				
Age (years)	50 (39–59)	49 (40–57)	50 (39–59)	0.975
Female, *n* (%)	25 (69.4)	12 (63.2)	13 (76.5)	0.615
Body mass index (kg/m^2^)	21.4 (18.5–23.1)	21.6 (20.5–24.1)	20.0 (18.0–22.4)	0.232
Current or past smoker, *n* (%)	6 (16.7)	5 (26.3)	1 (5.9)	0.232
**Inflammatory myopathy subtype**				
Dermatomyositis, *n* (%)	31 (86.1)	19 (100)	12 (70.6)	0.039
Polymyositis, *n* (%)	3 (8.3)	0 (0)	3 (17.6)	0.191
Others^*^, *n* (%)	2 (5.6)	0 (0)	2 (11.8)	0.418
**Autoantibody**				
Antinuclear antibody, *n* (%)	30 (83.3)	17 (89.5)	13 (76.5)	0.550
Anti-Ro (SSA) antibody, *n* (%)	4 (11.1)	3 (15.8)	1 (5.9)	0.680
Anti-La (SSB) antibody, *n* (%)	1 (2.8)	1 (5.3)	0 (0)	1.000
Anti-Jo1 antibody, *n* (%)	2 (5.6)	0 (0)	2 (11.8)	0.418
Anti-dsDNA, *n* (%)	2 (5.6)	1 (5.3)	1 (5.9)	1.000
Anti-Smith antibody, *n* (%)	1 (2.8)	0 (0)	1 (5.9)	0.955
Antinuclear ribonucleoprotein antibody, *n* (%)	1 (2.8)	0 (0)	1 (5.9)	0.955
**Maintenance immunosuppressive medication**				
Corticosteroid use, *n* (%)	31 (86.1)	16 (84.2)	15 (88.2)	1.000
Maintenance dose^†^ (mg/day)	18 (5–30)	20 (6–36)	16 (6–24)	0.514
Cyclosporin, *n* (%)	10 (27.8)	5 (26.3)	5 (29.4)	1.000
Methotrexate, *n* (%)	9 (25.0)	7 (36.8)	2 (11.8)	0.177
Azathioprine, *n* (%)	4 (11.1)	2 (10.5)	2 (11.8)	1.000
Tacrolimus, *n* (%)	3 (8.3)	2 (10.5)	1 (5.9)	1.000
Mycophenolate mofetil, *n* (%)	3 (8.3)	1 (5.3)	2 (11.8)	0.920
**Lung function (*****n*** **=** **16)**				
FVC (L)	1.6 (1.2–2.6)	2.3 (1.6–3.0)	1.2 (0.9–1.6)	0.011
FVC (%pred)	57.0 (40.7–67.3)	63.5 (54.5–73.8)	46.4 (32.9–58.3)	0.066
FEV_1_ (L)	1.4 (0.9–2.0)	2.0 (1.5–2.3)	0.9 (0.8–1.3)	0.002
FEV_1_ (%pred)	57.9 (41.6–66.7)	66.7 (61.2–73.9)	41.6 (34.7–52.0)	0.003
FEV_1_/FVC (%)	87.5 (77.9–93.1)	87.7 (77.9–93.1)	85.9 (70.8–91.7)	0.793
**Laboratory findings**				
LDH (IU/L) (*n* = 33)	331 (229–526)	358 (237–463)	312 (203–553)	0.871
CPK (IU/L) (*n* = 33)	130 (53–390)	130 (53–335)	147 (54–509)	0.652
ESR (mm/h) (*n* = 29)	51 (37–68)	49 (32–64)	57 (38–77)	0.266
CRP (mg/dL) (*n* = 33)	1.5 (0.4–2.2)	0.8 (0.1–1.8)	1.8 (0.9–3.8)	0.062
BNP (ng/mL) (*n* = 29)	54 (22–116)	34 (19–61)	102 (84–155)	0.020
**Comorbidity**				
Hypertension	11 (30.6)	4 (21.1)	7 (41.2)	0.281
Diabetes mellitus	8 (22.2)	6 (31.6)	2 (11.8)	0.236
Pulmonary hypertension	7 (19.4)	2 (10.5)	5 (29.4)	0.314

*Data were expressed as medians with interquartile ranges for continuous variables and numbers with percentages for categorical variables*.

* *Two other causes were mixed connective tissue disease with inflammatory myositis and systemic lupus erythematosus with inflammatory myositis*.

† *Dose is based on an equivalent dose of methylprednisolone*.

*AE, acute exacerbation; dsDNA, double-stranded deoxyribonucleic acid; SSA, Sjögren' syndrome type A; SSB, Sjögren' syndrome type B; FVC, forced vital capacity; FEV_1_, forced expiratory volume in 1 second; LDH, lactate dehydrogenase; CPK, creatinine phosphokinase; ESR, erythrocyte sedimentation rate; CRP, C-reactive protein; BNP, brain natriuretic peptide*.

The most common autoantibody was antinuclear antibody (83.3%), followed by anti-Ro (Sjögren' syndrome type A) antibody (11.1%), anti-double-stranded deoxyribonucleic acid (anti-dsDNA) (5.6%), and anti-Jo1 antibody (5.6%). The mainly prescribed maintenance immunosuppressive medication was corticosteroid (86.1%), followed by cyclosporin (27.8%), methotrexate (25.0%), azathioprine (11.1%), tacrolimus (8.3%), and mycophenolate mofetil (8.3%). However, there were no differences in the autoantibodies and maintenance immunosuppressive medications between the patients who developed non-infectious versus infectious AE. Patients with infectious AE had lower FVC (L) (*p* = 0.011), FEV_1_ (L) (*p* = 0.002), and the percentage-predicted value of FEV_1_ (*p* = 0.003) than those with non-infectious AE.

There were no significant intergroup differences in laboratory findings and comorbidities between patients with infectious AE and those with non-infectious AE, except for higher median brain natriuretic peptide (BNP) levels in patients with infectious AE than those with non-infectious AE (*p* = 0.020).

### Clinical Manifestations and Treatments of AE

As shown in Table 2, the median (IQR) of age at AE, time from admission to the BAL, and ICU length of stay was 53 (45–60) years, 10 (4–20) days, and 5 (0–17) h, respectively. Regarding the disease severity, the median (IQR) of the PF ratio and APACHE II score was 161 (89–241) mmHg and 10 (7–13), respectively. About two thirds of the patients required mechanical ventilation (66.7%). These observations and disease severities were similar between the two etiologies.

**Table 2:** Clinical manifestations and treatments of acute exacerbation (AE) of patients with myositis-related ILD.

	**Total (*n* = 36)**	**Non-infectious AE (*n* = 19)**	**Infectious AE (*n* = 17)**	***p*-value**
Age at AE (years)	53 (45–60)	53 (45–58)	53 (45–61)	0.924
ICU length of stay (day)	10 (4–20)	10 (5–18)	9 (2–20)	0.634
Time from admission to the BAL (hour)	5 (0–17)	10 (0–20)	2 (0–11)	0.188
**Disease severity**				
PF ratio (mmHg)	161 (89–241)	164 (87–201)	158 (105–300)	0.330
APACHE II scores	10 (7–13)	10 (7–13)	10 (6–14)	0.824
Mechanical ventilation, *n* (%)	24 (66.7)	13 (68.4)	11 (64.7)	1.000
**Radiologic feature**				
Number of lobe involvement, *n* (%)	2 (2–3)	2 (2–3)	2 (2–3)	0.242
Usual interstitial pneumonia, *n* (%)	8 (22.2)	6 (31.6)	2 (11.8)	0.305
Non-Specific interstitial pneumonia, *n* (%)	20 (55.6)	9 (47.4)	11 (64.7)	0.478
Organizing pneumonia, *n* (%)	5 (13.9)	4 (21.1)	1 (5.9)	0.406
**Laboratory findings**				
LDH (IU/L)	404 (281–642)	403 (289–560)	405 (281–708)	0.635
CPK (IU/L)	106 (45–327)	92 (57–262)	120 (42–993)	0.949
ESR (mm/h) (*n* = 33)	63 (35–77)	57 (39–73)	68 (35–83)	0.402
CRP (mg/dL) (*n* = 35)	5.0 (1.6–10.6)	5.0 (1.6–7.1)	6.5 (1.4–20.9)	0.643
BNP (ng/mL) (*n* = 31)	116 (77–306)	89 (48–132)	242 (104–470)	0.009
**Medication**				
Corticosteroid dose^†^ (mg/day)	63 (31–750)	125 (48–750)	50 (25–250)	0.212
IVIG, *n* (%)	7 (19.4)	4 (21.1)	3 (17.6)	1.000
Cyclophosphamide, *n* (%)	3 (8.3)	2 (10.5)	1 (5.9)	1.000
Mycophenolate mofetil, *n* (%)	1 (2.8)	1 (5.3)	0 (0.0)	1.000
Cyclosporin, *n* (%)	11 (30.6)	8 (42.1)	3 (17.6)	0.219

† *Dose is based on an equivalent dose of methylprednisolone*.

*AE, acute exacerbation; ICU, intensive care unit; BAL; bronchoalveolar lavage; PF, arterial oxygen partial pressure to fractional inspired oxygen; APACHE, acute physiology and chronic health evaluation; LDH, lactate dehydrogenase; CPK, creatinine phosphokinase; ESR, erythrocyte sedimentation rate; CRP, C-reactive protein; BNP, brain natriuretic peptide; IVIG, intravenous immunoglobulin*.

The most common radiologic feature was non-specific interstitial pneumonia (55.6%), and the median number of lobes involved was 2 [IQR, 2–3]. The patients with infectious AE showed higher median BNP levels than those with non-infectious AE [242 (IQR, 104–470) vs. 89 (IQR, 48–132) ng/ml], but there were no differences in radiological features or other laboratory findings between patients with non-infectious AE and those with infectious AE.

The main treatment for AE of myositis-related ILD was systemic corticosteroids. The median methylprednisolone equivalent dose of corticosteroids was 63 (IQR, 31–750) mg/day. The most common adjunctive treatment was cyclosporin (30.6%), followed by intravenous immunoglobulin (19.4%), cyclophosphamide (8.3%), and mycophenolate mofetil (2.8%). Although the median corticosteroid dose was 2.5-fold lower in infectious AE than non-infectious AE [50 (IQR, 25–250) vs. 125 (IQR, 48–750) mg/day], this difference was not significant (*p* = 0.212). In addition, there were no statistical significances in the use of adjunctive treatments between the groups.

### Infectious Etiologies and Mortality

**Figure 2:**
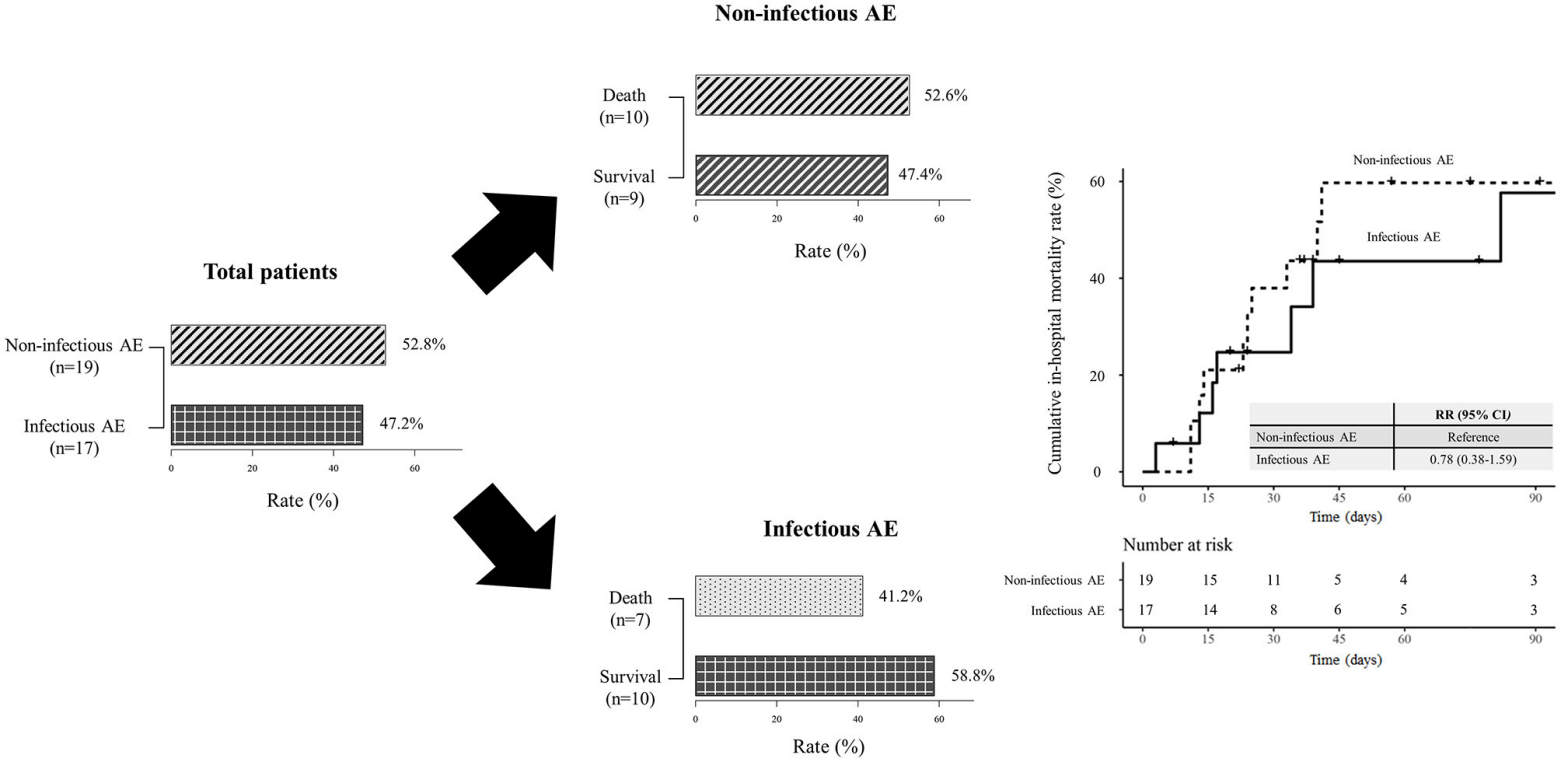
The rate and mortality of AE of myositis-related interstitial lung disease according to the etiology. AE, acute exacerbation; RR, risk ratio; CI, confidence interval.

Among the 36 patients with AE of myositis-related ILD, 17 were diagnosed with infectious AE. The identified organisms were *Streptococcus pneumonia* (*n* = 2), *Pseudomonas aeruginosa* (*n* = 3), anaerobes (*n* = 3), cytomegalovirus (*n* = 6), herpes simplex virus (*n* = 1), and *pneumocystis jirovecii* (*n* = 4). The overall in-hospital mortality rate of AE of myositis-related ILD was 47.2%. Although the mortality rate in patients with infectious AE was lower (41.2%) than in those with non-infectious AE (52.6%), this difference was not statistically significant (*p* = 0.724). A survival analysis showed no significant difference in mortality in patients with infectious AE and those versus non-infectious AE (*RR* = 0.78, 95% *CI* = 0.38–1.59) (Figure 2).

## Discussion

In this study, we evaluated the treatment outcomes in patients with myositis-related ILD by etiologies. Our study showed that survival was not significantly different between the patients with myositis-related ILD in whom the etiologies (i.e., infectious vs. non-infectious) were retrospectively evaluated.

While respiratory infections are a known etiology for the AE of myositis-related ILD, the exact causes or mechanisms for non-infectious AE are unknown (2). Liang et al. found that the increased myositis activity at the time of admission was a risk factor for AE of myositis-related ILD (5), suggesting that AE may result from an intrinsic acceleration of the underlying disease. Supporting this view, anti-Melanoma Differentiation-Associated gene 5 (anti-MDA5) antibody, an autobody of myositis, was shown to be associated with rapidly progressive ILD (15). Intrinsic biological dysfunction caused by increased levels of myositis-related cytokines, which makes patients more vulnerable to external stimuli, could be the other possible mechanism (16). However, there is still a lack of sufficient understanding of the mechanism of developing AE in patients with myositis-related ILD. Therefore, further studies are needed on this issue.

In clinical practice, distinguishing infectious etiologies from non-infectious etiologies for AE of myositis-related ILD is important to provide an appropriate treatment. However, unfortunately, our study results showed that it is very hard to distinguish infectious AE from non-infectious AE based on the clinical manifestation since there were no differences in radiologic features and laboratory findings by etiology. In addition, despite the prompt attempts to unveil the infectious etiologies for AE (all patients underwent BAL within 24 h of admission) and appropriate antibiotic use, our study showed that infectious causes had a similar risk of in-hospital mortality compared with non-infectious AE of myositis-related ILD. Accordingly, as in AE of IPF (17), our study results indicate that respiratory infections are important causes of AE in myositis-related ILD (8). A similar definition and classification of AE of ILD used in IPF may also be used in patients with myositis-related ILD. Thus, it is suggested that clinicians consider infections as a high priority in patients with AE of myositis-related ILD. We would like to suggest more rapid diagnostic approaches to identify potential pathogens and immediate treatment for possible infectious causes to improve treatment outcomes in myositis-related ILD patients with infectious AE.

The treatment outcome of non-infectious AE of myositis-related ILD was very poor in this study, showing more than 50% of mortality within 90 days after admission despite using high-dose of systemic corticosteroid with/without adjunctive treatments. The treatment of non-infectious AE of myositis-related ILD has not been standardized, and there have been no randomized clinical trials that have compared the efficacy of these treatments (18). Nevertheless, corticosteroid therapy has been the cornerstone of treatment for AE of myositis-related ILD, and intravenous immunoglobulin has been used as an adjunctive treatment (19). The poor outcomes of corticosteroid-based treatment in our study may indicate that we need alternative treatments for patients who are refractory to corticosteroids. Fortunately, depending on the better understanding of the mechanism of some endotypes of myositis-related ILD (e.g., anti-synthetase syndrome and anti-MDA5 positive myositis-related ILD), novel immunologic agents have shown some potential to be used for this purpose (20). For example, a Janus kinase (JAK) inhibitor, tofacitinib, significantly increased the survival rate of patients with refractory rapidly progressive myositis-related ILD whose treatment failed with high-dose steroids and adjunctive drugs (21). Another study showed that Rituximab improved the lung function in patients with refractory anti-synthetase syndrome (22). A recent study proposed that biologics [e.g., anti-interferon α, anti-interferon α receptors antibodies, anti-interleukin 12/23, anti-interleukin-6, or other anti-B cell therapy (e.g., ibrutinib)], and antifibrotic agents (e.g., nintedanib and pirfenidone), might be promising (20). However, since there is a paucity of data on the efficacy of these novel agents for the treatment of AE in patients with myositis-related ILD, more evidence is needed.

Our study has the strength that the largest number of patients with AE of myositis-related ILD was evaluated. In previous studies, due to the low incidence of IIM, myositis-related ILD was commonly studied with other connective tissue disease-related ILD (23). However, there are some limitations in our study. First, our study was retrospective and performed in a single center. Second, some important myositis-associated antibodies, such as anti-MDA5 antibody, were not assessed because these tests were unavailable in Korea. Thus, we could not evaluate the impact of anti-MDA5 antibody on the presentation of AE and treatment outcomes. Since previous studies have shown that patients with anti-MDA5 have rapidly progressive characteristics of ILD (15, 20), anti-MDA5 might be associated with developing non-infectious AE in our study. To overcome these limitations, future studies, including a comprehensive evaluation of myositis-associated antibodies, are necessary.

In conclusion, our study showed that infectious AE is an important cause of mortality in patients with myositis-related ILD, showing a similar mortality rate for non-infectious AE. More rapid diagnostic approaches to identify potential pathogens and immediate antibiotic coverage for possible infectious causes would be likely to be important to improve treatment outcomes. Our study suggests that the detailed phenotyping or endotyping of non-infectious AE of myositis is needed to improve treatment outcomes.

## Data Availability Statement

The raw data supporting the conclusions of this article will be made available by the authors, without undue reservation.

## Ethics Statement

The studies involving human participants were reviewed and approved by the Institutional Review Board (IRB) of Hanyang University Hospital (IRB Number: HYUH 2020-04-056-002). Written informed consent for participation was not required for this study in accordance with the national legislation and the institutional requirements.

## Author Contributions

HL: conceptualization. SC and SK: formal analysis and writing—original draft. TP, DP, J-YM, S-HK, TK, and HY: review and editing. JS: supervision and validation. All authors read and approved the final manuscript.

## Funding

This work was supported by the National Research Foundation of Korea (NRF) grant funded by the Ministry of Science, Information, and Communications Technologies (MSIT) (NRF-2020R1F1A1070468 and NRF-2021M3E5D1A01015176). This work was also supported by the Korea Medical Device Development Fund grant funded by the Korea government (the Ministry of Science and ICT, the Ministry of Trade, Industry and Energy, the Ministry of Health & Welfare, the Ministry of Food and Drug Safety) (Project Numbers: 1711138447 and KMDF_PR_20200901_0214).

## Conflict of Interest

The authors declare that the research was conducted in the absence of any commercial or financial relationships that could be construed as a potential conflict of interest.

## Publisher's Note

All claims expressed in this article are solely those of the authors and do not necessarily represent those of their affiliated organizations, or those of the publisher, the editors and the reviewers. Any product that may be evaluated in this article, or claim that may be made by its manufacturer, is not guaranteed or endorsed by the publisher.

## References

[1] Lundberg IE Miller FW Tjärnlund A Bottai M. Diagnosis and classification of idiopathic inflammatory myopathies. J Intern Med. (2016) 280:39–51. 10.1111/joim.12524 27320359 PMC5021058

[2] Connors GR Christopher-Stine L Oddis CV Danoff SK. Interstitial lung disease associated with the idiopathic inflammatory myopathies: what progress has been made in the past 35 years? Chest. (2010) 138:1464–74. 10.1378/chest.10-0180 21138882

[3] Vencovský J Alexanderson H Lundberg IE. Idiopathic inflammatory myopathies. Rheum Dis Clin. (2019) 45:569–81. 10.1016/j.rdc.2019.07.006 31564297

[4] Barba T Fort R Cottin V Provencher S Durieu I Jardel S. Treatment of idiopathic inflammatory myositis associated interstitial lung disease: a systematic review and meta-analysis. Autoimmun Rev. (2019) 18:113–22. 10.1016/j.autrev.2018.07.013 30572131

[5] Liang J Cao H Ke Y Sun C Chen W Lin J. Acute exacerbation of interstitial lung disease in adult patients with idiopathic inflammatory myopathies: a retrospective case-control study. Front Med. (2020) 7:12. 10.3389/fmed.2020.00012 32083087 PMC7005087

[6] Fujisawa T Suda T Nakamura Y Enomoto N Ide K Toyoshima M. Differences in clinical features and prognosis of interstitial lung diseases between polymyositis and dermatomyositis. J Rheumatol. (2005) 32:58–64. Available online at: https://www.jrheum.org/content/32/1/58.long 15630726

[7] Shappley C Paik JJ Saketkoo LA. Myositis-Related interstitial lung diseases: diagnostic features, treatment, and complications. Curr Treatm Opt Rheumatol. (2019) 5:56–83. 10.1007/s40674-018-0110-6 31984206 PMC6980290

[8] Collard HR Ryerson CJ Corte TJ Jenkins G Kondoh Y Lederer DJ. Acute exacerbation of idiopathic pulmonary fibrosis. An international working group report. Am J Respir Crit Care Med. (2016) 194:265–75. 10.1164/rccm.201604-0801CI 27299520

[9] Collard HR Moore BB Flaherty KR Brown KK Kaner RJ King TE Jr. Acute exacerbations of idiopathic pulmonary fibrosis. Am J Respir Crit Care Med. (2007) 176:636–43. 10.1164/rccm.200703-463PP 17585107 PMC2094133

[10] Molyneaux PL Maher TM. The role of infection in the pathogenesis of idiopathic pulmonary fibrosis. Eur Respir Rev. (2013) 22:376–81. 10.1183/09059180.00000713 23997064 PMC9487348

[11] Azadeh N Limper AH Carmona EM Ryu JH. The role of infection in interstitial lung diseases: a review. Chest. (2017) 152:842–52. 10.1016/j.chest.2017.03.033 28400116 PMC7094545

[12] Leclair V Lundberg IE. New myositis classification criteria-what we have learned since bohan and peter. Curr Rheumatol Rep. (2018) 20:18. 10.1007/s11926-018-0726-4 29550929 PMC5857275

[13] Graham BL Steenbruggen I Miller MR Barjaktarevic IZ Cooper BG Hall GL. Standardization of spirometry 2019 update. An official American thoracic society and European respiratory society technical statement. Am J Respir Crit Care Med. (2019) 200:e70–88. 10.1164/rccm.201908-1590ST 31613151 PMC6794117

[14] Choi JK Paek D Lee JO. Normal predictive values of spirometry in Korean population. Tuberc Respir Dis. (2005) 58:230–42. 10.4046/trd.2005.58.3.230

[15] Li L Wang Q Wen X Liu C Wu C Yang F. Assessment of anti-MDA5 antibody as a diagnostic biomarker in patients with dermatomyositis-associated interstitial lung disease or rapidly progressive interstitial lung disease. Oncotarget. (2017) 8:76129–40. 10.18632/oncotarget.19050 29100298 PMC5652692

[16] Gono T Kaneko H Kawaguchi Y Hanaoka M Kataoka S Kuwana M. Cytokine profiles in polymyositis and dermatomyositis complicated by rapidly progressive or chronic interstitial lung disease. Rheumatology. (2014) 53:2196–203. 10.1093/rheumatology/keu258 24970922

[17] Invernizzi R Molyneaux PL. The contribution of infection and the respiratory microbiome in acute exacerbations of idiopathic pulmonary fibrosis. Euro Respir Rev. (2019) 28:190045. 10.1183/16000617.0045-2019 31285290 PMC9488861

[18] Morisset J Johnson C Rich E Collard HR Lee JS. Management of myositis-related interstitial lung disease. Chest. (2016) 150:1118–28. 10.1016/j.chest.2016.04.007 27102182

[19] Bakewell CJ Raghu G. Polymyositis associated with severe interstitial lung disease: remission after three doses of IV immunoglobulin. Chest. (2011) 139:441–3. 10.1378/chest.10-0360 21285059

[20] Hervier B Uzunhan Y. Inflammatory myopathy-related interstitial lung disease: from pathophysiology to treatment. Front Med. (2019) 6:326. 10.3389/fmed.2019.00326 32010700 PMC6978912

[21] Kurasawa K Arai S Namiki Y Tanaka A Takamura Y Owada T. Tofacitinib for refractory interstitial lung diseases in anti-melanoma differentiation-associated 5 gene antibody-positive dermatomyositis. Rheumatology. (2018) 57:2114–9. 10.1093/rheumatology/key188 30060040

[22] Fasano S Gordon P Hajji R Loyo E Isenberg DA. Rituximab in the treatment of inflammatory myopathies: a review. Rheumatology. (2017) 56:26–36. 10.1093/rheumatology/kew146 27121778

[23] Huapaya JA Wilfong EM Harden CT Brower RG Danoff SK. Risk factors for mortality and mortality rates in interstitial lung disease patients in the intensive care unit. Eur Respir Rev. (2018) 27:180061. 10.1183/16000617.0061-2018 30463873 PMC9489107

